# Editorial

**Published:** 1844-10-19

**Authors:** 


### THE MEDICAL EXAMINER.

PHILADELPHIA, OCT. 19, 1844.

### CLINICAL LECTURES AND REPORTS.

The many favorable expressions we have heard from our subscribers, in reference to this department of the *Examiner*, satisfies us of its utility; and accordingly it will continue to be with us a prominent object of attention. The lectures of Sir Benjamin Brodie, are admitted by every one to be exceedingly rich and valuable, not only because of his vast experience, but from the clearness with which the subjects are presented, the aptness of his illustrations, and the plain common sense views which characterize his remarks on all occasions. Next to this eminent surgeon, Mr. Liston stands prominently in England, as well as in the estimation of the profession in this country: we have therefore collected some of the lectures delivered by him at one of the great London Hospitals, which, after we shall have concluded those of Sir Benjamin Brodie, will appear in our columns. By an accident, (which it is not necessary to explain) one of Mr. Liston's lectures has fallen into the printers' hands prematurely, and hence it appears in the present number. Although they will not be forthcoming for a time, the present sample will afford to our readers a foretaste of what are to come.

The season is approaching when the clinical courses in our own Colleges and Hospitals will be resumed with their wonted spirit. From these sources, also, we hope to draw valuable materials for our pages.

### PROVINCIAL MEDICAL AND SURGICAL ASSOCIATION, (ENGLAND.)

The Provincial Medical and Surgical Journal of August 14th, is filled with the proceedings of this spirited association. It appears that the twelfth anniversary was held at Northampton, on Wednesday, August 7th, and Thursday, August 8th, at which there were *one hundred and fifty members present*, from various parts of the Kingdom, many of whom we recognize as amongst the most eminent members of the profession. The greatest harmony and good feeling

.evailed, and much important business was transacted. Among the proceedings, we notice the following in relation to one of our own countrymen who was then in England:

"Dr. Forbes moved, and Dr. Cowan seconded—

"That Thomas D. Mütter, M. D., of Philadelphia, Professor of Surgery in Jefferson Medical College, be appointed an Honorary Corresponding Member of the Provincial Medical and Surgical Association."

